# Aging, Metabolic, and Degenerative Disorders: Biomedical Value of Antioxidants

**DOI:** 10.1155/2018/2098123

**Published:** 2018-04-10

**Authors:** Mohamed M. Abdel-Daim, Nadia I. Zakhary, Lotfi Aleya, Simona G. Bungǎu, Raghvendra A. Bohara, Nikhat J. Siddiqi

**Affiliations:** ^1^Pharmacology Department, Faculty of Veterinary Medicine, Suez Canal University, Ismailia 41522, Egypt; ^2^Department of Ophthalmology and Micro-Technology, Yokohama City University, Yokohama, Japan; ^3^Cancer Biology Department, National Cancer Institute, Cairo University, Cairo 11796, Egypt; ^4^Chrono-Environment Laboratory, Bourgogne Franche-Comté University, UMR CNRS 6249, 25030 Besançon Cedex, France; ^5^Pharmacy Department, Faculty of Medicine and Pharmacy, University of Oradea, Oradea, Romania; ^6^Centre for Interdisciplinary Research, D. Y. Patil University, Kolhapur 416006, India; ^7^Department of Biochemistry, College of Science, King Saud University, Riyadh 11495, Saudi Arabia

Oxidative stress is a common aetiological factor in aging, metabolic diseases, and degenerative disorders. The body contains an endogenous antioxidant system to help neutralize reactive oxygen species and mitigate oxidative damage. Every living cell survives when there is a balance between the oxidative stress generated and the counter antioxidant system present. Once this harmony is disrupted, the generated stress loads on the cell and starts to exert pathological, metabolic, and degenerative effects. Antioxidants have been proven to ameliorate drug toxicity [1], carcinogenesis [2], and neurodegenerative processes [3, 4]. In this special issue, several articles have proposed different molecular mechanisms to mitigate oxidative stress and prevent aging, metabolic, and degenerative disorders.

For example, S. M. Ahmed et al. searched and analyzed previous animal studies to develop a conclusive systematic review discussing the use of different types of mesenchymal stromal cells (MSCs) for the treatment of acute and chronic pancreatitis and pancreatic fibrosis. They concluded that bone marrow and umbilical cord MSCs were the most frequently administered cell types. In addition, they did not recommend clinical trials to investigate the use of MSCs as therapy for pancreatitis due to the insufficiency of published data.

In cardiovascular research, S. I. Khan et al. evaluated the effects of febuxostat and allopurinol on a rat model of ischemia-reperfusion (IR) injury and concluded that febuxostat had more potent protective activities than allopurinol against IR injury by inhibiting apoptosis (MAPK) and inflammation (NF-*κ*Bp65/TNF-*α*) pathways. In another study, N. M. Al-Rasheed et al. investigated the potential cardioprotective effect of simvastatin on a diabetic cardiomyopathy (DCM) rat model and suggested that simvastatin ameliorates DCM by attenuating inflammation, oxidative stress, and apoptosis, induced by hyperglycemia and hyperlipidemia.

In the field of diabetes research, B. Assefa et al. investigated the mechanisms underlying insulin-like growth factor binding protein-2- (IGFBP-2-) stimulated glucose uptake in adipocytes and concluded that the potentiating effects of IGFBP-2 on 3T3-L1 adipocyte GU are independent of its binding to IGF-1 and might occur, which was mediated through the activation of PI3K/Akt, AMPK/TBC1D1, and PI3K/PKC*ζ*/*λ*/GLUT-4 signaling. In addition, H. M. A. Abdelrazek et al. evaluated the protective role of black seed oil (NSO) active constituent in streptozotocin-induced diabetic rats and showed that NSO improved oxidative stress, hepatic glycogen storage, and pancreatic islet insulin secretion.

Another study in oncology, performed by E. A. Toraih et al., examined the expression of miR-34a and miR-11 of its bioinformatically selected target genes and proteins to test their potential dysregulation in renal cell carcinoma tumorigenesis and cancer progression. Moreover, Z. You et al. evaluated the alleviating role of fermented papaya extracts (FPEs) in estrogen- and progestogen-induced mammary gland hyperplasia via their antioxidant activities and inhibition of DNA damage.

T.-Y. Song et al. showed the possible protective effects of ergothioneine and hispidin on methylglyoxal-induced neuronal cell hyperglycemic damage in rat pheochromocytoma cells through inhibition of oxidative stress and the NF-*κ*B transcription pathway, which adds to the rich neuroscience literature. Further, B. Pietrucha et al. compared the antioxidant status and major lipophilic antioxidants in patients with ataxia-telangiectasia (AT) and Nijmegen breakage syndrome (NBS) and confirmed the irregularities in redox homeostasis, and reduction of coenzyme Q10 in AT and NBS patients could be used as potential diagnostic tools in these diseases. In addition, M. S. Fawzy et al. examined the expression of longevity-related transcriptional factors (SOX2, OCT3/4, and NANOG) to evaluate them as diagnostic tools as well as to treat glioblastoma multiforme.

Finally, A. B. Abdel-Naim et al. isolated rutin isolated from *Chrozophora tinctoria* along with other five flavonoids and found that rutin enhanced bone cell proliferation and ossification markers using human osteosarcoma cell lines (SAOS-2 and MG-63). We hope that the readers of this special issue will find it enlightening about the potential benefits of antioxidants, which may help them fill the knowledge gaps in the prevention and treatment of aging, metabolic, and degenerative disorders.

*Mohamed M. Abdel-Daim* *Nadia I. Zakhary* *Lotfi Aleya* *Simona G. Bungǎu* *Raghvendra A. Bohara* *Nikhat J. Siddiqi*
